# Erectile Dysfunction and Permanent Bladder Areflexia Following Montivipera Bornmuelleri Snakebite

**DOI:** 10.7759/cureus.17968

**Published:** 2021-09-14

**Authors:** Maytham Al-Musawi, Hani Chanbour, Jad El Masri, Rabih Awad, Khalil Armash

**Affiliations:** 1 Urology, Beirut Arab University, Beirut, LBN; 2 Medicine, Faculty of Medicine, Lebanese University, Beirut, LBN; 3 Neuroscience Research Center, Faculty of Medicine, Lebanese University, Beirut, LBN; 4 Urology, Faculty of Medicine, Lebanese University, Beirut, LBN; 5 Urology, Nini Hospital, Tripoli, LBN

**Keywords:** montivipera bornmuelleri, venom, weakness, areflexic bladder, snakebite

## Abstract

Lebanese viper (*Montivipera bornmuelleri) *is a well-known venomous snake that inhabits the middle eastern region, famous for its hypercoagulable properties and cytotoxic effects. The daunting neurological dysfunction caused by other snakebites remains unreported in this particular species of middle eastern vipers. We describe a case of a 31-year-old male patient who presented with right leg *Montivipera bornmuelleri *snakebite. He initially suffered from decreased level of consciousness and generalized tonic-clonic seizure, followed by right leg motor weakness, as well as urinary incontinence and dribbling, with complete erectile dysfunction and anesthesia over the groin area, while preserving the sensation at the level of the anus. Brain and spinal magnetic resonance imaging, electromyography, and laboratory tests were all normal. Urodynamic study showed complete bladder areflexia. The patient initiated intermittent bladder catheterization, with no improvement of his symptoms. To our knowledge, this is the first case of neurological dysfunction brought by the famous Lebanese venomous snake.

## Introduction

Snakebite is a worldwide medical issue. Around 5.4 million persons are bitten every year, leading to 81,000 to 138,000 annual deaths [1]. Most patients presenting with snakebites come from a rural background, reflecting a low socioeconomic status [2].

Lebanon has around 25 species of snakes, three of which belong to the family of vipers. Lebanon viper (*Montivipera bornmuelleri*) is a venomous snake that dwells in Syria and the highest mountains of Lebanon [3]. This snake grows to a maximum length of 75 cm, having a tail consisting of 7-10% of its total length [4]. It belongs to the kingdom Animalia, division Chordata, class Reptilia, order Squamata, family Viperidae, and genus Montivipera [4].

This snake’s venom affects the coagulation cascade (through the intrinsic pathway), either by inducing clot formation or hindering it [5]. It also decreases blood pressure via its vasorelaxant property. As suggested by previous studies, this venom has an immunomodulatory effect and a cytotoxic effect that may be useful in cancer treatment [6].

Despite the well-established association between snakebites and neurotoxicity, no study has suggested a clear relation between *Montivipera bornmuelleri’s* venom and neurological sequelae. Cases of irreversible neurological symptoms remain scarce, and cases of urological complications following snakebites have never been reported in the literature.

Erectile dysfunction, bladder areflexia, and delayed irreversible motor and sensory symptoms are described in a young Lebanese man, following a snakebite by the well-known *Montivipera bornmuelleri* viper.

## Case presentation

**Figure 1:**
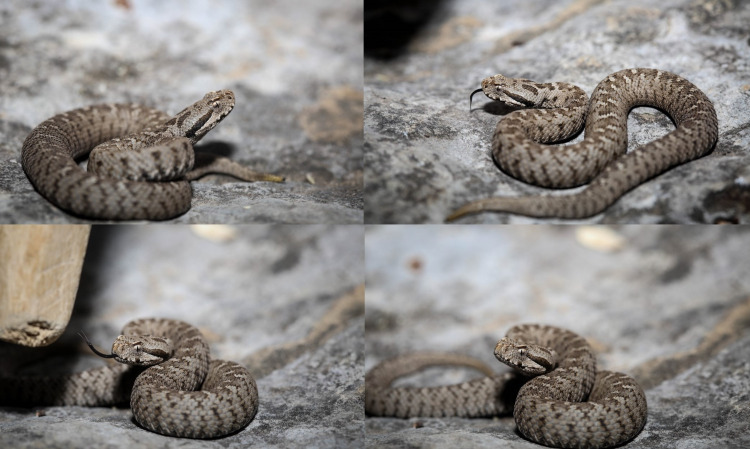
The famous Lebanese viper Montivipera bornmuelleri. The image is captured by Dr. Rabih Awad (coauthor) at the site where the snake attacked the patient, the night following the injury. It is described by the villagers - and later on by the patient - as the snake responsible for the injury.

A 31-year-old Caucasian male patient, previously healthy, was bitten by a poisonous snake on his right leg. He identified the snake as *Montivipera bornmuelleri* (Figure 1). Patient was fully conscious when he presented one hour after the bite to a peripheral hospital.

Upon arrival, the patient developed a decreased level of consciousness and a subsequent generalized tonic-clonic seizure. Glasgow Coma Scale was 11/15. IV diazepam was administered at site. Vitals were stabilized.

Diffuse edema and redness were found at the site of the snakebite, the wound was cleaned with normal saline and antiseptic. The patient was admitted to the intensive care unit for monitoring, foley was inserted, and he was given human tetanus immunoglobulin (1 mL 250 IU) and four vials of antivenom (40 mL). Complete blood counts, creatine phosphokinase, liver enzymes, creatinine, magnesium, calcium, prothrombin time (PT), partial thromboplastin time (PTT), and international normalized ratio (INR) were all within normal range.

The next day, the patient was clinically and hemodynamically stable and was transferred to the regular floor. Neurological examination showed right lower limb motor weakness 3/5 both proximally and distally. Whereas the sensory examination demonstrated total deficits in pain, temperature, and light touch in the right leg with normal deep tendon reflexes.

The patient was discharged two days later, on a combination of vitamin B complex 300 mg two times daily. Three days after discharge, the patient started to have urinary incontinence and dribbling, with complete erectile dysfunction and anesthesia over the groin area, with preserved sensation at the level of the anus. Electromyography (EMG) of the right leg was done and was completely normal, gabapentin was added, given 300 mg at day one, increased daily to reach 1800 mg at day six and thereafter. The patient was poorly followed up due to financial issues. Brain and spinal MRI was completely normal. However, cerebrospinal fluid (CSF) analysis wasn’t approved by the insurance plan.

**Figure 2:**
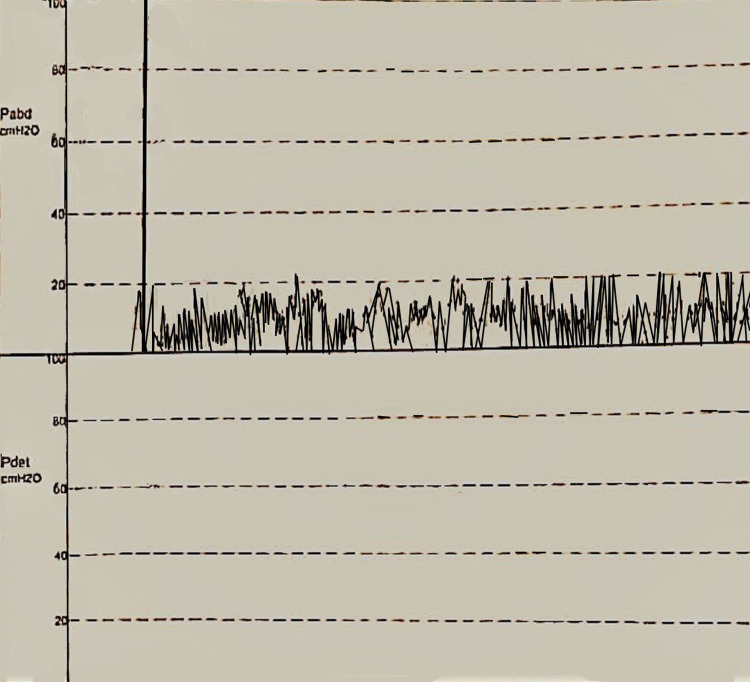
Urodynamic study after infusion of normal saline, showing an increased intraabdominal pressure reaching 20 cmH20, without any concomitant increase in the detrusor muscle pressure, interpreted as detrusor muscle atony, reflecting an areflexic bladder. Pabd: intraabdominal pressure, Pdet: detrusor muscle pressure Pressure in cmH2O.

To control his urinary symptoms, he used a condom catheter and decreased his water intake. Three months later, he returned to the neurologist with no improvement of his urinary symptoms and sensation at the groin area, but there was only mild improvement of his right leg motor power and no sensory change. Electromyogram was repeated and showed no evidence of change. Right lower limb venous duplex ultrasound was done and there were no signs of deep venous thrombosis, superior thrombophlebitis, and soft tissue edema. The patient was scheduled for urodynamic study, which showed increased functional bladder capacity, no uninhibited bladder contraction, no sensation to void, and post-void residue of 700 mL, as detrusor pressure remained null (Figure 2). During the placement of the foley catheter, there was no sensation. Diagnosis of the complete areflexic bladder was concluded. The patient was advised to carry on intermittent catheterization two times daily, upon subsequent follow-up, no change in the patient’s symptomatology was noted.

## Discussion

To our knowledge, this is the first report to correlate snakebites with urological symptoms, in particular, erectile dysfunction and bladder areflexia. Furthermore, irreversible unilateral leg weakness and paresthesia, as well as permanent penile anesthesia, haven’t been reported in the previous literature. Although no previous study showed any correlation between *Montivipera bornmuelleri* snake venom and neurological symptoms, this relation is not unfathomable, as reversible neurotoxicity is common among snake bites. For instance, snakebites causing focal peripheral nerve damage were previously reported. Our normal MRI findings have basically ruled out any major ischemic or hemorrhagic complications that could explain the ensuing neurological deterioration. The envenomization following the initial injury releases cytotoxic enzymes that activate pro-inflammatory mechanisms, which leads to nerve necrosis [7]. As a consequence, a myriad of neurological manifestation ensues the venom release, such as ptosis, ophthalmoplegia, limb weakness, respiratory failure, palatal weakness, neck muscle weakness, and delayed sensory neuropathy [8,9]. The greater majority of these symptoms are usually of acute onset, and rapidly reversible after anti-venom injection, lasting for few hours to few days [9]. A study conducted in France on *Vipera aspis* aspis (V.a.a), a snake that descends from the same ancestry of *Montivipera bornmuelleri* showed that V.a.a’s contains neurotoxins that cause severe neurological signs and cephalic muscle paralysis [10].

Viper’s venom contains 65 enzymatic protein families such as serine proteases, phospholipases A2 (PLA2), and metalloproteases III [11]. These potent enzymes can interfere with the coagulation cascade, the normal homeostatic system and tissue repair. In addition, the venom has antimicrobial effect, working against Gram-positive and Gram-negative bacteria, mainly working against *Staphylococcus aureus* and *Morganella morganii* strains [11].

PLA2 is a mammalian body component and is suggested to bind to its natural receptor in the human body, causing neurotoxicity [11]. Snake neurotoxins endowed with PLA2 activity enter neurons and attach to mitochondria, inducing a shape change within nerve terminals. These neurotoxins lead to the release of Ca^2+^, causing a defective synaptic transmission, provoking motor and sensory abnormalities [12]. The right leg weakness and paresthesia experienced by our patient can be explained by a neurotoxic effect at the site of the bite. The peculiar property of irreversibility after anti-venom administration, even after a year of the initial bite, suggests a permanent neurotoxic effect to the nerve ending, that wasn’t detected on EMG.

Regarding the delayed urological symptoms, with the concomitant anesthesia of the genital area, and the combined autonomic/somatic sensory and motor deficits in the detrusor muscle and the penile shaft, propose delayed neuropathic changes. The latter can originate from autoimmune processes, such as Guillain-Barre syndrome [13] or irreversible neurotoxicity caused by antibodies directed to the snake’s venom or antivenom [14]. The delayed presentation of the patient may have played a major role in the sequalae that rose after the snake bite. The only urological complication that was manifested after a snakebite belongs to viper’s family (Russell’s viper), which led to priapism, which subsided after administrating antivenom [15]. Despite being completely unrelated to our case, this report may suggest a direct venom inoculation of the penile shaft, with immediate toxicity. Management of snakebites can be tricky, the use of vacuum extractor, and transporting the patient to the nearest medical facility proved efficient to prevent delayed damage and wound infections. Traditional measures like applying tourniquets, incision and suction, and ice therapy are highly ineffective [16]. After hospitalization, systemic reaction, laboratory findings, and physical examination should be taken into consideration to determine whether there is envenomization or not [16]. If no symptoms appeared after six hours, the patient is assured that no envenomization was present, while there is a need of antivenom in case any symptoms occurred [17]. Regarding the use of prednisone after snakebite, a study on dogs bitten by a *Vipera berus* did not support its routine use following the bite [18]. Steroids are beneficial only if the patient develops allergic reaction and anaphylaxis against the antivenin [19].

Regarding neurotoxicity, there are some reports that showed recovery from neuromuscular paralysis without the use of antivenom, while other stated antivenom administration before recovery [8]. Antivenom can only be effective if given early to neutralize circulating venom before binding to its target, as it cannot neutralize bound venom [20].

## Conclusions

This case presents unique delayed symptomatology of *Montivipera bornmuelleri* snakebite. Areflexic bladder and erectile dysfunction, along with irreversible neuropathic/neurotoxic sensory and motor symptoms following the famous Lebanese viper should shed the light on improving and building new facilities close to rural areas where these bites are more common, to help implement an early and aggressive treatment, in order to prevent rare but feared sequelae.
